# Multidisciplinary Approach to Spinal Cord Stimulation for Persistent Spinal Pain Syndromes: A 65-Month Integrated Data Collection From the Belgian Neuro-Pain® Real-World Data Register

**DOI:** 10.1155/prm/7880611

**Published:** 2025-08-04

**Authors:** Lisa Bernaerts, Ella Roelant, Maarten Moens, Huynh Giao Ly, Jean-Pierre Van Buyten, Bart Billet, Bart Bryon, Martine Puylaert, Turgay Tuna, Maureen Malone, Tom Theys, Anne Berquin, Johan Vangeneugden, Guy Hans

**Affiliations:** ^1^Multidisciplinary Pain Center, Antwerp University Hospital (UZA), Edegem, Belgium; ^2^Laboratory of Pain Research, ASTARC, University of Antwerp (UA), Wilrijk, Belgium; ^3^Clinical Trial Center, Antwerp University Hospital (UZA), Edegem, Belgium; ^4^Department of Neurosurgery, University Hospital Brussels, Brussels, Belgium; ^5^Department of Implants and Invasive Medical Devices, National Institute for Health and Disability Insurance (NIHDI), Brussels, Belgium; ^6^Multidisciplinary Pain Center, AZ Vitaz, Sint Niklaas, Belgium; ^7^Multidisciplinary Pain Center, AZ Delta, Roeselare, Belgium; ^8^Multidisciplinary Pain Center, AZ Turnhout, Turnhout, Belgium; ^9^Multidisciplinary Pain Center, Ziekenhuis Oost-Limburg, Genk, Belgium; ^10^Multidisciplinary Pain Center, Hôpital Erasme, ULB, Brussels, Belgium; ^11^Multidisciplinary Pain Center, AZ Klina, Brasschaat, Belgium; ^12^Department of Neurosurgery, University Hospitals Leuven, Leuven, Belgium; ^13^Department of Physical and Rehabilitation Medicine, Cliniques Universitaires UCL, St Luc, Brussels, Belgium; ^14^Department of Neurosurgery, AZ Sint Maarten, Mechelen, Belgium

**Keywords:** chronic pain, long-term follow-up, neuromodulation, real-world data, registry, spinal cord stimulation

## Abstract

**Background:** Spinal cord stimulation (SCS) serves as a treatment option for neuropathic pain conditions. Despite its widespread use and technological advancements over the last decade, the long-term efficacy of SCS remains a topic of debate. Consequently, there is an increasing demand for real-world, long-term data regarding its effectiveness.

**Material and Methods:** In 2018, the Belgian government launched a nationwide platform to monitor all SCS therapies. Five and a half years after its start, a full data extraction was conducted. In the present study, we update the findings of Bernaerts et al. (2024) from the 3-week trial period and the long-term follow-up of patients with persistent spinal pain syndrome and focus on the completion rates of the follow-ups and the battery lifetime of the implantable pulse generators (IPGs).

**Results:** Findings indicate that “yellow flags” or psychological variables can be confirmed as significant predictors of recovery and satisfaction following the trial. Additionally, these yellow flags were able to predict long-term disability. Analysis revealed that patients who completed the follow-up module displayed more active and less passive coping strategies for their pain, along with lower levels of illness anxiety prior to the trial's start, better physical and psychological functioning, and greater recovery and satisfaction with the trial's outcomes. However, adherence to the chronic follow-up module declined over time. Moreover, we investigated the battery life of both rechargeable and nonrechargeable batteries across various indication types. The real-world dataset indicated no significant differences in battery lifetime between rechargeable and nonrechargeable IPGs for each indication type.

**Conclusions:** The long-term outcomes of neuromodulation are intricate and influenced by various factors. Data extracted from the Neuro-Pain® registry increasingly enable us to identify confounding factors and predictors of treatment success with greater precision.

**Trial Registration:** ClinicalTrials.gov identifier: NCT06835868

## 1. Introduction

Spinal cord stimulation (SCS) is an invasive yet reversible option for managing persistent spinal pain syndrome. Although there is significant scientific evidence [1–3], long-term real-world data (RWD) on SCS results are still limited. Since 2018, relevant data on all Belgian patients who have received invasive neuromodulation have been collected in the interactive register, Neuro-Pain®. This centralized real-world database, combined with ongoing patient monitoring, provides valuable insights into the long-term effectiveness and safety of SCS on a national scale.

This study aims to update previously reported results [4] after 5.5 years of data collection. More specifically, the study seeks to provide an update on the findings of the 3-week trial period and long-term follow-up of patients with persistent spinal pain syndrome. In addition, the relationship between psychological factors, “yellow flags,” and outcome measures will be described. Furthermore, as observed in our previous study [4], compliance with completing chronic follow-up questionnaires over prolonged periods seems to decrease significantly over time. Therefore, to further investigate this phenomenon, we aimed to compare the patients who completed the chronic follow-up and those who dropped out. In addition, we aimed to examine the battery lifetimes of both rechargeable and nonrechargeable implantable pulse generators (IPGs) across various clinical indications. The IPG's longevity directly impacts long-term costs to the healthcare payers and to society in general. The longer the device lasts, the lower the total cumulative costs will be, especially concerning device replacement costs [5]. A previous retrospective study of Medicare claims demonstrated that the clinical longevity is similar between rechargeable and nonrechargeable batteries [6]. However, an earlier study indicated that the median longevity of IPGs was significantly greater for rechargeable devices compared to nonrechargeable SCS devices [7].

## 2. Methods

### 2.1. Recruitment of Patients

All patients were treated at Belgian multidisciplinary pain centers. Notably, Belgium has 35 recognized multidisciplinary pain centers. Since January 2018, all patients who started receiving SCS and those requiring battery replacements have been enrolled in the Neuro-Pain® platform. The registry does not include PNS and multifidus stimulators. DRG stimulation is included but utilizes a different pathway; therefore, these results are not included in the current discussion. Patients provided informed consent for the use of their data to evaluate the efficacy of the technique. The creation of the Neuro-Pain® register was approved by the National Chamber of Social Security and Health on Data Protection (https://www.ksz-bcss.fgov.be/nl/page/de-kamer-sociale-zekerheid-en-gezondheid).

### 2.2. First Implant Procedure for an IPG

**Figure 1:**
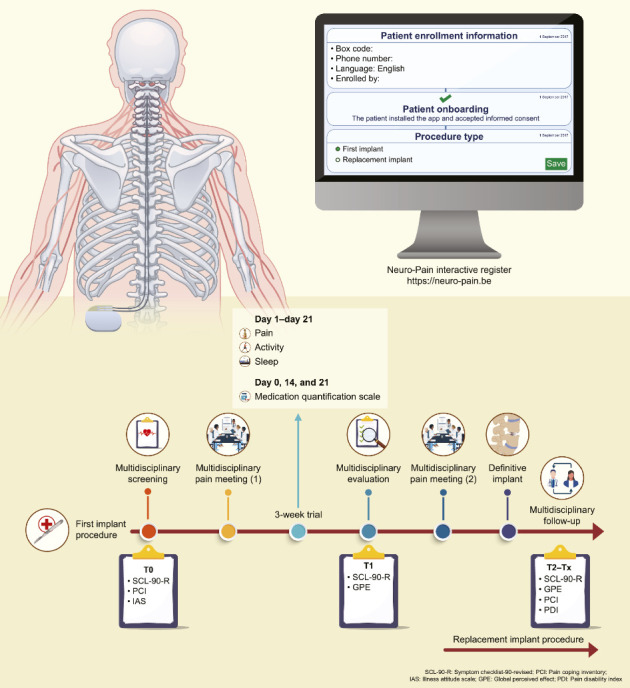
Graphical representation of the timelines for a first implant procedure (first line) and a replacement implant procedure (second line) within the Neuro-Pain® platform.

Eligible patients underwent multidisciplinary screening (T0) (Figure 1) to determine their suitability as candidates for SCS. Subsequently, they were referred for a psychological pre-evaluative assessment, which included two consultations with a pain psychologist and a series of questionnaires evaluating general psychological functioning, pain coping strategies, and illness anxiety. Psychiatric consultation was sought only when psychiatric conditions (e.g., clinical depression or addiction) were identified. Patients were invited to enroll in a web-based platform, Neuro-Pain®, where they completed additional questionnaires. After gathering the required data, a multidisciplinary pain meeting was convened involving a pain specialist, neurosurgeon, and pain psychologist (psychiatrist). In this meeting, the team decided whether the patient met the medical and psychological criteria for SCS and if multidisciplinary consent was granted to initiate the trial. Once approved, the patient underwent a 3-week trial period with implanted electrodes and an external impulse generator. Within the Neuro-Pain® platform, the patient maintained a diary detailing their pain levels, activity, and sleep quality. Medication usage was reviewed by the attending physician prior to the start of trial therapy and updated by the patient on Days 14 and 21. Medical and psychological evaluations were conducted following trial completion (T1). Afterward, patients were requested to complete questionnaires regarding their psychological functioning, sense of recovery, and treatment satisfaction. The trial period results were discussed in a subsequent multidisciplinary pain meeting. Significant reductions in pain and pain medication, improved sleep quality, and increased activity levels demonstrated a successful trial. Implantation of an IPG was carried out after securing multidisciplinary consent. Following definitive implantation, patients were asked to complete questionnaires every 6 months (T2–T11). While it is highly recommended to evaluate a patient's overall functioning with an implant, online multidisciplinary follow-up is not mandatory. The physician completed a chronic follow-up module based on the patient's pain score, sleep quality, and adjustment to pain medication usage.

### 2.3. Replacement Implant Procedure

Since 2018, patients who previously received SCS treatment and required an IPG replacement have also been registered on the Neuro-Pain® platform. The new IPG device was implanted after consent was obtained from the multidisciplinary team. Additionally, patients were invited to complete the questionnaires every 6 months (T2–T11).

### 2.4. Screening Measures

Patients completed a pre-evaluative psychological inventory that included the Symptom Checklist-90-Revised (SCL-90-R), Pain Coping Inventory (PCI), and Illness Attitude Scale (IAS). Each patient underwent two psychological pre-evaluative screening consultations with a pain psychologist. During the 3-week trial period, patients filled out a daily survey on pain, activity, and sleep. The attending physician completed the patient's Medication Quantification Scale (MQS-IIIR) on Day 0, and patients updated their medication status on Days 14 and 21 [8, 9]. After the trial, they completed a postevaluative psychological questionnaire that included the SCL-90-R and General Perceived Effect (GPE), and all patients participated in a psychological postevaluation consultation. Pre- and postevaluations were mandatory for all patients per the royal decree. Following this, patients were invited to complete chronic follow-up questionnaires every 6 months after definitive implantation, although this was not mandatory. These follow-up questionnaires comprised the SCL-90-R, GPE, PCI, and Pain Disability Index (PDI). Questionnaires were completed on the patient-specific online platform Neuro-Pain®, available in Dutch, French, and German, allowing patients to select their preferred language. Bernaerts et al. (2024) provided comprehensive information about developing and implementing the secure interactive register, Neuro-Pain®, and the associated questionnaires [4].

### 2.5. Statistical Analysis

Data collection through the Neuro-Pain® platform started on February 14, 2018. The data extraction on which this report is based occurred on May 30, 2023. No data cleaning was performed. For 366 records, no additional information was available beyond the initial demographic data; therefore, these records were excluded from further analyses.

A total of 8846 records from 7210 different patients were included in this analysis. One patient could have multiple records (e.g., one initial implant procedure and two subsequent replacements). The following patient data were recorded in the database: date of birth, age (at the time of data extraction), and sex. Nineteen patients were excluded from calculating the mean and standard deviation (SD) of age because an estimated age of less than 8 was considered a typing error. The characteristics of the included 8846 records are described below. This dataset is referred to as the full dataset in the following analyses.

For 3894 patients, the initial implant procedure was recorded in the database. For 3165 patients of these, 3201 records included pre-evaluation, trial, and postevaluation data. In instances of duplicates, the most complete record for each patient was prioritized. For nine duplicates, the completion rate was identical across both records, and the first entry was selected. This dataset of 3165 patients with initial implant data was used for further analysis and referred to as the subdataset in the following analyses.

The number of physicians and patients who completed the follow-up questionnaires every 6 months after the definitive battery implantation varied. Patients could only complete their questionnaires after their physician submitted the previous follow-up questionnaire. Except for the first follow-up questionnaire, these questionnaires were promptly sent to both doctors and patients. For each patient in the full dataset, the number of follow-ups completed by the physician (for pain, sleep, or medication) and those achieved by the patient (PDI, SCL-90-R, PCI, or GPE) were counted. To calculate a percentage, the denominator excluded those patients for whom the follow-up time did not allow for the specified number of follow-ups.

For longitudinal analysis, we analyzed the subdataset differently [4] by adjusting the timing of the questionnaires based on the actual completion date. The start of the follow-up module was influenced by several factors, including the accurate completion date of the surgery, whether the physicians fulfilled their responsibilities, and when the patients submitted their questionnaires. We adjusted the timing of the questionnaires as described below.

The T2 questionnaires were completed between Days 181 and 274 (6–9 months); the T3 questionnaires took place between Days 275 and 457 (9–15 months); the T4 questionnaires were filled out between Days 458 and 640 (15–21 months); the T5 questionnaires were completed from Days 641 to 823 (21–27 months); the T6 questionnaires were completed between Days 824 and 1006 (27–33 months); the T7 questionnaires were filled out between Days 1007 and 1189 (33–39 months); the T8 questionnaires were completed between Days 1190 and 1372 (39–45 months); the T9 questionnaires were completed from Days 1373 to 1555 (45–51 months); the T10 questionnaires were filled out between Days 1556 and 1738 (51–57 months); and finally, the T11 questionnaires were completed between Days 1739 and 1921 (57–63 months).

Two free-text boxes (replacements for another diagnosis and the early termination of the trial) were quantified and analyzed. These data points are presented in a tabular format, with each row representing a specific patient's procedure. The clinical diagnosis of pain was entered as free text, and symptom categories (neuropathy, algoneurodystrophy [complex regional pain syndrome, CRPS], posttraumatic pain syndrome, polyneuropathy, postoperative pain, sciatica, headache, backache, facial palsy, and pancreatitis) were retrieved. If the reason for discontinuing the procedure was “infection,” this could be obtained from another free-text field. These categories were represented in binary format, indicating the presence or absence of symptoms. The detection method utilized a dictionary-based approach, in which the source data were iteratively scanned to identify a list of triggers for each symptom. This method ensured the detection of linguistic variations, negation terms, and the language used within the free-text field by the patients. The symptoms were manually evaluated using a programmable Python script, guaranteeing the repeatability of the extended dataset.

In the subdataset, the group of patients who discontinued the procedure after the first multidisciplinary pain meeting was compared with those who continued the procedure, utilizing an unpaired *t*-test (with equal or unequal variances) or the Mann–Whitney test, as appropriate. The *p* values were adjusted for multiple testing using the Bonferroni–Holm correction, which was implemented only for three subscales of the PCI Active Coping, three subscales of the PCI Passive Coping, eight subscales of the Dutch version of the SCL-90-R, nine subscales of the French and German versions of the SCL-90-R, and two subscales of the IAS.

During the trial period, patients responded to three daily diary questions: pain intensity, activity, and sleep. Weekly summaries of these values were generated in the subdataset, and a linear mixed model was employed to analyze the three measurements, treating time as a fixed effect and subjects as random effects. If a significant time effect was detected, pairwise *post hoc* comparisons among the three time points were performed using Tukey's correction for multiple comparisons. This same modeling approach was applied to the MQS-IIIR measurements taken on Days 0, 14, and 21. Other outcomes, including SCL-90-R, GPE, PDI, and PCI, were assessed multiple times and analyzed similarly, utilizing all available measurements in the subdataset, with time as a fixed effect and subjects as random effects. Significant time effects triggered *post hoc* tests, comparing consecutive time points. The Bonferroni–Holm correction was applied to the *p* values associated with the time effects for the subscales and *post hoc* comparisons.

To investigate the hypothesis that yellow flags predict lower recovery and satisfaction after the trial period, a logistic regression model was fitted to the subdataset with recovery and satisfaction as binary outcomes (a score above four is classified as outcome 1, while a score of 4 or less is classified as outcome 0) and yellow flags as predictors. The Bonferroni–Holm correction for multiple tests was conducted on the subscales as previously described. A similar logistic regression model examined whether yellow flags predicted decreased functioning in the long term. As in [4], 6 months and 1.5 years after definitive battery implantation are considered, but now 2.5 years has also been added. Therefore, disability was categorized into two groups: above (Outcome 1) or below (Outcome 0) the median PDI (51 at 6 months, 52 at 1.5 years, and 53 at 2.5 years). Alternative statistical analyses were performed for the sensitivity analysis. An ordinal regression model was fitted with recovery and satisfaction as ordinal outcome measures (1–7) and yellow flags as predictors, while a linear regression model used disability as a continuous outcome measure (0–100) to investigate whether yellow flags could predict lower functioning after 6 months and 1.5 years. These alternative statistical analyses are excluded from the scope of this article for the convenience of readers.

The group of patients who completed the first follow-up (after 6 months) is compared with those who did not in terms of pre-and postevaluative screening measures using an unpaired *t*-test (with equal or unequal variances as appropriate) in the subdataset. Descriptive statistics of battery lifetime, categorized by indication type and battery type, are reported for the replacement records in the full dataset. A Kaplan–Meier curve is displayed for battery lifetime by indication type and battery type. Battery lifetime is compared between indication types within battery types and between battery types per indication type using a linear mixed model to account for records from the same patient.

Statistical significance was set at *p* < 0.05. Significant *p* values are shown in bold, and analyses were performed using R Version 4.1.2 [10]. Only the results that differ from those in the initial analyses [4] will be described in this manuscript.

## 3. Results

### 3.1. Participants

The average age of patients in the full dataset was 58.3 years (SD, 11.6 years), with ages ranging from 21 to 93 years. Among the participants, there were 4437 females (61.5%), 2771 males (38.4%), and two individuals whose gender was unspecified (0.03%). Of these cases, 55.2% pertained to replacement procedures, while 44.8% were related to primo-implant procedures. In the primo-implant category, 90.1% of records indicated a diagnosis of failed back surgery syndrome (FBSS), while 9.9% indicated the presence of a failed neck surgery syndrome (FNSS). Regarding replacement procedures, 80.4% of patients were diagnosed with FBSS, 7.7% with FNSS, 0.8% with CRPS (these results will not be discussed in this study), and 11.1% (a total of 542 records) were classified under “other diagnoses.” This subgroup was further analyzed using a textbox where physicians could enter additional diagnoses relevant to SCS initiation. Results revealed that within these records, 310 patients were treated for “neuropathy,” 42 for “algoneurodystrophy (CRPS),” 75 for “post-trauma,” six for “polyneuropathy,” 49 for “postoperative,” 30 for “sciatica,” 13 for “headache,” eight for “back pain,” six for “pancreatitis,” two for “facialgia,” and 10 for “phantom pain.” The native language of the patients—Dutch, French, or German—was determined by their choice of language on the initial questionnaire. Among the patients, 74.3% selected Dutch, 25.4% opted for French, and 0.3% chose German as their preferred language to complete the questionnaires.

Regarding follow-up questionnaires to be completed every six months after definitive device implantation, physicians did not complete a single follow-up for 3688 patients. They completed one follow-up for 1270 patients. In total, 4624 patients did not complete any follow-up. Additionally, 1364 patients completed one follow-up. On average, each patient completed 0.73 questionnaires.  Table 1 provides an overview of the number of completed follow-ups using a different approach to calculating the completion rates compared to Bernaerts et al. [4], as described in the Materials and Methods section.

### 3.2. Were Yellow Flags Predictive of Less Recovery and Less Satisfaction After Trial (T1)?

In the previous publication by Bernaerts et al. [4], no significant predictor was found for recovery. However, logistic regression analyses of the current subdataset showed that PCI Total Active Coping was a significant predictor of recovery. For satisfaction, several significant predictors were confirmed in the current subdataset compared to Bernaerts et al.: PCI Total Passive Coping, PCI Retreating, PCI Worrying, and IAS Illness Anxiety. Additionally, some other significant predictors were identified: PCI Total Active Coping, PCI Distraction, and SCL-90-R Depression (see Table 2 for detailed information on *p* values, OR, and 95% CI).

### 3.3. Were Yellow Flags Predictive of Lower Functioning After 6 Months (T2), 1.5 Years (T4), or 2.5 Years (T6)?

Logistic regression analyses of the subdataset showed similar significant predictors as in [4] for disability after 6 months (T2): PCI Total Passive Coping (*p* < 0.001, OR 1.021, 95% CI [1.014, 1.028]), PCI Retreating (*p* < 0.001, OR 1.014, 95% CI [1.008, 1.019]), PCI Worrying (*p* < 0.001, OR 1.011, 95% CI [1.006, 1.017]), PCI Resting (*p* < 0.001, OR 1.014, 95% CI [1.009, 1.019]), IAS Illness Behavior (*p* < 0.001, OR 1.130, 95% CI [1.093, 1.169]), SCL-90-R Agoraphobia^a^ (*p*=0.003, OR 1.015, 95% CI [1.006, 1.025]), SCL-90-R Depression^a^ (*p* < 0.001, OR 1.016, 95% CI [1.009, 1.023]), SCL-90-R Somatic Complaints^a^ (*p* < 0.001, OR 1.020, 95% CI [1.011, 1.029]), SCL-90-R Insufficiency^a^ (*p* < 0.001, OR 1.022, 95% CI [1.015,1.030]), SCL-90-R Sensitivity^a^ (*p*=0.014, OR 1.014, 95% CI [1.004,1.023]), SCL-90-R Sleep Problems^a^ (*p*=0.036, OR 1.006, 95% CI [1.001,1.010]), SCL-90-R Psychoneuroticism^a^ (*p* < 0.001, OR 1.022, 95% CI [1.012,1.033]), SCL-90-R Somatization^b^ (*p*=0.010, OR 1.051, 95% CI [1.019,1.085]), SCL-90-R Anxiety^b^ (*p*=0.005, OR 1.060, 95% CI [1.024, 1.098]), SCL-90-R Global Severity Index^b^ (*p*=0.001, OR 1.895, 95% CI [1.285, 2.843]), and MQS Day 0 (*p*=0.010, OR 1.015, 95% CI [1.004, 1.026]). But also some other, as in [3] significant predictors are found namely: SCL-90-R Depression^b^ (*p*=0.014, OR 1.033, 95% CI [1.011, 1.055]), SCL-90-R Phobic Anxiety^b^ (*p*=0.004, OR 1.072, 95% CI [1.031, 1.118]) and SCL-90-R Psychoticism^b^ (*p*=0.047, OR 1.064, 95% CI [1.015, 1.119]).

After 1.5 years (T4), compared to [4] besides PCI Resting (*p* < 0.001, OR 1.033, 95% CI [1.021, 1.045]), several other significant predictors of disability are indicated: PCI Total Passive Coping (*p* < 0.001, OR 1.029, 95% CI [1.016, 1.044]), PCI Retreating (*p*=0.020, OR 1.012, 95% CI [1.002, 1.023]), PCI Worrying (*p*=0.006, OR 1.015, 95% CI [1.005, 1.026]), IAS Illness Behavior (*p*=0.001, OR 1.121, 95% CI [1.052, 1.196]), SCL-90-R Insufficiency^a^ (*p*=0.005, OR 1.022, 95% CI [1.009, 1.036]), and MQS Day 0 (*p*=0.002, OR 1.036, 95% CI [1.013, 1.060]).

After 2.5 years (T6), the analyses showed two remaining significant predictors of disability: PCI Total Passive Coping (*p*=0.008, OR 1.023, 95% CI [1.006, 1.041]) and PCI Resting (*p*=0.012, OR 1.018, 95% CI [1.006, 1.032]). ^a^ and ^b^ are the Dutch and French/German versions of the SCL-90-R, respectively. All *p* values were corrected for multiple testing.

### 3.4. Who Completed the Chronic Follow-Up Questionnaire?

After device implantation, the patients completed chronic follow-up modules. The completion of such a module by the patient depends on the physician's completion of the follow-up module, so we only analyzed the first follow-up module (after 6 months). This initial module was accessible to both the patient and the physician. Following multiple corrections for testing, the PCI Pain transforming, PCI total passive coping, PCI Retreating, PCI Resting, IAS Illness anxiety, and behavior of the pre-evaluative screening measures showed significant differences between patients who completed the follow-up module and those who did not in the subdataset (Table 3). Additionally, all subscales of the psychological postevaluative measures (SCL-90-R and GPE) demonstrated significant differences between the patients who completed the follow-up module and those who did not, even after corrections for multiple comparisons (see Table 4).

### 3.5. Battery Lifetime Across Indication Types

**Figure 2:**
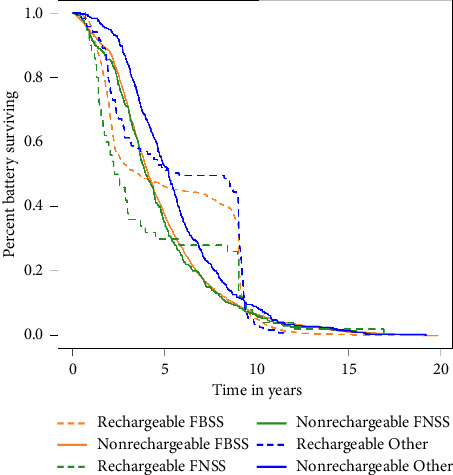
Kaplan–Meier curve of battery survival time in years comparing battery types and indication types.

Table 5 presents the descriptive statistics on battery lifetime expressed in days and years. The indication type and battery type (rechargeable cell versus nonrechargeable or primary cell batteries) were distinguished.  Figure 2 presents the Kaplan–Meier plot of battery survival time in years, categorized by battery type and indication type. We notice a gradual decline for nonrechargeable primary cell batteries, where the survival distribution of the battery lifetime for FBSS and FNSS appears very similar. For the indication “Other,” nonrechargeable batteries, however, seem to last longer. Comparing rechargeable to nonrechargeable batteries, we see that the curves cross at 4 (FBSS) or 5 years (FNSS, “Other”). Where originally nonrechargeable batteries had longer lifetimes, the situation now shifts, as few rechargeable batteries fail between 4 and 5 years, and even fewer fail between 5 and 9 years. After 9 years, a significant drop in the curve is observed, indicating that most rechargeable batteries have reached, by that time, the end of their lifespan.

A linear mixed model was applied to account for duplicate entries (1058 observations for 1051 patients with a rechargeable type of impulse generator and 3340 observations for 2695 patients with a nonrechargeable IPG type). Overall, we observed significant differences in battery lifetime among the three indication types (FBSS, FNSS, and “Other,” *p*=0.045) for the rechargeable battery and even more pronounced differences for the nonrechargeable battery (FBSS, FNSS, and “Other,” *p*=0.002). Pairwise *post hoc* comparisons of the three groups indicated a significant difference in battery lifetime between FBSS and “Other” (*p* < 0.001) and FNSS and “Other” (*p* < 0.001) for nonrechargeable batteries, as well as between FNSS and “Other” (*p*=0.035) for rechargeable batteries (see Table 6). No significant differences in battery lifetime were found between rechargeable and nonrechargeable batteries for each of the three indication types.

## 4. Discussion

### 4.1. Participants

Following repeated activation of the chronic follow-up module, the number of patients completing long-term follow-up declined. This creates an opportunity to mandate the completion of this chronic follow-up module or to develop engaging experiences further to optimize long-term monitoring of treatment outcomes in future studies. Enhancing physician involvement through an online follow-up module is also crucial.

### 4.2. Yellow Flags Are Predictive of Less Recovery and Less Satisfaction After Trial (T1)

This updated analysis confirms that yellow flags are predictive of recovery levels and satisfaction after the trial. Logistic regression analysis indicated the predictive value of active pain-coping strategies on the degree of recovery following the trial. Additionally, both active and passive pain-coping strategies, along with depression and illness anxiety, were predictive of satisfaction levels after the trial. Feelings of recovery and satisfaction appear to be linked to other predictors.

This updated analysis thus confirms that pain-coping strategies highlighted how recovered and satisfied patients perceived the trial's outcome. Patients who employed passive pain-coping strategies reported lower levels of satisfaction after the trial. In contrast, patients who utilized more active pain-coping strategies experienced greater recovery and satisfaction following the trial. A patient's thoughts, feelings, and behaviors in response to pain significantly influence their improvement and satisfaction after a neuromodulation trial. Besides coping, feelings of depression and attitudes and feelings regarding illness predict patient satisfaction with trial results.

### 4.3. Yellow Flags Are Predictive of Lower Functioning After 6 Months (T2), 1.5 Years (T4), and 2.5 Years (T6)

This updated analysis includes an extra follow-up period at 2.5 years (T6) postimplant. This updated analysis confirms the predictive value of yellow flags 6 months after battery implantation but also adds longer-term predictive value. As previously demonstrated in our national real-world database, yellow flags are predictive of disability levels both 1.5 years and 2.5 years after initial assessment. Pain-coping strategies significantly predict a patient's disability level up to 2.5 years postimplantation. Patients who relied more on passive pain-coping techniques reported feeling more disabled over time. Specifically, those who utilized rest as a passive coping method experienced increased feelings of disability for up to 2.5 years after the procedure. Furthermore, this analysis shows that illness behaviors can predict a patient's sense of disability at 6 months and 1.5 years following a permanent implant, with patients displaying greater illness behaviors also feeling more disabled in the long run.

### 4.4. Patients Who Completed the Chronic Follow-Up

After the implantation of the permanent device, patients engaged with the chronic follow-up modules. Statistical analyses revealed significant differences between those who completed the first follow-up module and those who did not. Patients who completed the first module demonstrated notably higher scores on the active coping subscale of pain transformation and significantly lower scores on the passive coping subscales of retreating and resting prior to the trial's onset. These patients could sometimes envision their pain as less intense, recognizing that others also faced difficulties. Additionally, they appeared to withdraw less from stimuli and engaged in less resting behavior. Furthermore, those who completed the first follow-up module reported significantly lower scores on illness anxiety and illness behavior before the trial began, indicating reduced concerns about their health. These patients also had notably lower scores across all SCL-90-R subscales and total scores, alongside significantly higher scores on the GPE subscales of Recovery and Satisfaction following the trial. As a result, patients who participated in the follow-up module exhibited lower levels of physical and psychological dysfunction after the trial, feeling more recovered and satisfied. We found that these patients employed a more active approach to managing their pain, utilized fewer passive coping strategies, and reported lower illness anxiety before the trial. They also demonstrated improved physical and psychological functioning, as well as greater satisfaction with the trial outcomes. The “satisfied” patients expressed feeling improved post-treatment and demonstrated higher compliance. In contrast, the “less satisfied” patients withdrew from the first follow-up, potentially representing a more vulnerable group who may benefit most from ongoing support postimplantation.

### 4.5. Battery Longevity Across Different Indication Types

Our analysis of battery lifetime in SCS revealed no significant differences in battery longevity between rechargeable and nonrechargeable batteries across the three indication types. When comparing clinical longevity among different indication types, we observed that patients treated with SCS for indications other than persistent spinal pain syndrome type 2 (FBSS and FNSS) experienced longer longevity with rechargeable IPGs compared to patients with FNSS. Patients with these “Other” indications also had greater longevity for nonrechargeable primary cell IPGs than patients with FBSS and FNSS. On average, an FNSS patient depleted a rechargeable battery 1.54 years sooner than a patient with an “Other” indication. Similarly, an FBSS patient utilized a nonrechargeable battery 0.82 years less than a patient with an “Other” indication, and an FNSS patient drained a nonrechargeable battery 0.97 years sooner than a patient with an “Other” indication. These data demonstrated a significant difference in battery longevity between patients with persistent spinal pain syndrome type 2 and those with an “Other” indication, favoring the latter. Our findings are consistent with a recent publication on the retrospective evaluation of Medicare claims [6]. However, the average lifespan of IPGs in our registry is shorter than reported in several other studies [6, 7]. This could be due to several factors, such as the inclusion of batteries that are still operational and have not yet reached their end-of-life. This could lead to an underestimation of the battery's longevity. Technical issues could also result in earlier replacement. In a real-life setting with diverse clinical indications, there is a greater need for stimulation adjustments in patients, which could result in a sooner depletion of batteries. Nevertheless, it is essential to note that our registry has the longest follow-up among the various battery types, indicating that in real-world practice, IPG longevity is indeed less than what manufacturers claim. These findings will be further clarified when more IPGs within the real-world database reach their end of life in the following years.

### 4.6. Comparison With Previous Findings

This study updates a previous analysis by Bernaerts et al. [4] by incorporating an additional 10 months of data, resulting in 1542 new records and 1040 additional patients. We found no substantial differences between our previous and current analyses. Several trends identified earlier persisted in this analysis. Two key findings emerged. First, our initial analysis indicated that yellow flags did not predict the extent of recovery post-trial. However, an active coping style is now identified as a predictor of improved perception of recovery after the trial. Previously, a passive coping style appeared to correlate with post-trial satisfaction; in contrast, our current study suggests that both active and passive coping styles can be predictive. Furthermore, depression (assessed via the SCL-90-R) and illness anxiety (measured by the IAS) have become predictive in this analysis. Hence, our latest findings highlight the relevance of yellow flags as predictors for recovery perceptions and satisfaction following SCS trials. Second, while our first analysis identified several predictors of disability after 6 months of SCS treatment, the current analysis confirms these and adds additional subscales for the French and German versions of the SCL-90-R. Although only a few patients completed the questionnaires in these languages, the results gradually expanded. In our initial analysis, only a few psychological variables were found to predict disability after 1.5 years. In this most recent analysis, many more variables appear to be predictive. Passive coping in general, and retreating and worrying more specifically, along with illness behavior and medication use on Day 0, determine the level of disability after 1.5 years of permanent implant. Passive coping even remains predictive after 2.5 years. In conclusion, as the dataset grows, some findings are confirmed and proven to be robust, while other trends continue to emerge and become more apparent.

This study emphasizes the challenges patients face regarding compliance. Only 23.7% of patients completed the first follow-up module, and 22.1% had it filled out by a physician. Our analysis revealed that patients who completed this module tended to use more active coping strategies and fewer passive ones for managing pain. They also exhibited lower illness anxiety before beginning the trial, along with better physical and psychological functioning, as well as greater recovery and satisfaction with trial outcomes. Those who were “less satisfied” chose to drop out after the first follow-up. Limited research provides context for these findings. Hirsch et al. explained that patients often distinguish between the quality of care, which involves interpersonal relationships, and the quality of treatment, which focuses on outcomes [11, 12]. This distinction may help clarify the paradoxical connection between patient satisfaction and symptom relief, as patients can report high satisfaction despite limited pain alleviation. Our results illustrated this divergence in recovery and satisfaction concerning yellow flags. Hirsch et al. examined the link between satisfaction and compliance with treatment recommendations [11, 12].

Oosterhaven et al. examined how catastrophizing affects patient dropout rates in an interdisciplinary pain management program [13]. While this study focused on participants who dropped out during treatment, it showed that beliefs and coping styles were important predictors of adherence. The research found that dropouts were significantly more likely to catastrophize compared to those who finished the program. The Pain Catastrophizing Scale (PCS) measured catastrophizing behavior, with domain scores for Helplessness (*p*=0.001) and Rumination (*p*=0.009), as well as the total PCS score (*p*=0.001), indicating notable differences between the two groups. Additionally, our observations suggested that patients' overall constitution—covering coping mechanisms and well-being—at the start of treatment greatly influenced their compliance during ongoing follow-up. Therefore, a patient's perception and coping strategies regarding pain from the beginning of treatment are vital for maintaining treatment expectations.

### 4.7. Limitations, Evolutions, and Opportunities in the Use of the Centralized Interactive Register

The current data extraction has several limitations. First, patients may have provided socially desirable answers to complete the trial and receive a permanent implant, which could have introduced bias into the results and conclusions. This issue needs to be addressed during the data collection process. Second, completing the chronic follow-up questionnaires was optional but highly recommended; however, compliance decreased over time, making conclusions about the implants' long-term performance unreliable. In this study, there seemed to be greater clarity regarding whether the questionnaires could be completed and the reliance on physicians for their completion. Including the actual date of questionnaire submission aimed to enhance the accuracy of the analysis. We also assessed whether patients completed their first follow-up questionnaire; however, follow-up data were absent for 80% of patients. The analysis of patients who completed the first follow-up questionnaire indicated that they belong to the “more recovered and satisfied” group. Therefore, the interpretation of the available long-term analyses is somewhat questionable. Since we did not have outcome data for all patients (both recovered and less recovered), caution should be exercised when generalizing our results. Furthermore, as noted earlier, there were limitations, including (a) complications arising from the use of the SCL-90-R in three national languages; (b) the absence of baseline pretrial information on vital parameters such as pain, sleep, and activity; (c) the online registration process requiring basic computer skills that not all patients possessed; and (d) the required 21-day trial period, which could predispose patients to infections or technical failures.

Finally, one could question the validity of data collected from registries since they are merely observational and cannot establish cause and effect. However, we believe that the Neuro-Pain® register addresses many challenges of RWD by ensuring low heterogeneity and multimodality while maximizing data representativeness [14]. Our goal is not to establish causality; instead, we utilize the RWD from the register to address practical issues in SCS, employing techniques such as prediction, pattern recognition, and knowledge discovery. This report should be viewed as a RWD study that does not aim to establish causality (WOCO) [15].

## 5. Conclusion

The aims of this study were achieved in three ways. First, we provided an update on our initial results (Bernaerts et al., 2024) after 5.5 years of data collection. Compared to the previous report, we included 10 months of additional data, resulting in 1542 extra records and 1040 additional patients. We also provided an update on the 3-week trial and long-term follow-up of patients with persistent spinal pain syndrome. However, no major differences were observed between the two analyses. Some trends from the previous analyses continued, including the relationship between psychological factors, yellow flags, and outcome measures. The following hypotheses were investigated: “Are yellow flags predictive of less recovery and satisfaction after the trial?” and “Are yellow flags predictive of lower functioning after 6 months, 1.5 years, and 2.5 years?” Yellow flags appeared to be significant predictors of recovery and satisfaction after the trial, as well as long-term disability. These findings were confirmed using an expanded dataset. Second, the study demonstrated that compliance with chronic follow-up questionnaires decreased over time. We analyzed patients who completed the chronic follow-up. Our analysis indicated that patients who completed the follow-up module tended to adopt more active and less passive coping strategies for their pain, had lower levels of illness anxiety before the trial, and exhibited better physical and psychological functioning, as well as greater recovery and satisfaction with the trial results. The “less satisfied” patients, for whom follow-up was essential, withdrew during the first follow-up. Third, we examined the lifetimes of rechargeable and nonrechargeable batteries across different indication types. The analysis of the clinical longevity of IPGs in a real-world dataset showed no major differences in battery lifetime between rechargeable and nonrechargeable batteries for the three indication types. There was a difference in battery lifetime between a patient with persistent spinal pain syndrome type 2 and a patient with an “Other” indication, favoring the latter.

Recent advances in neuromodulation have improved treatment outcomes for patients with various chronic pain syndromes [16–19]. However, during periods of budget constraints, every treatment must meet the four objectives of value-based healthcare: enhancing patient experience, improving population health, reducing costs, and improving provider experience. To achieve these objectives, government agencies and scientific organizations should support the creation of national (or international) RWD registries. In the fast-changing landscape of healthcare practice, RWD have the potential to provide reliable new evidence, driving healthcare advancements, influencing reimbursement policies, and cost-effectively enhancing patient care.

## Figures and Tables

**Table 1:** Number of chronic follow-up questionnaires filled in by the physician and by the patient.

Number of follow-ups	Physician, *n* (%)	Patient, *n* (%)
0	3688 (64.2)	4624 (80.4)
1	1270 (22.1)	1364 (23.7)
2	607 (11.5)	551 (10.4)
3	433 (9.1)	277 (5.8)
4	346 (8.2)	180 (4.3)
5	307 (8.5)	107 (3.0)
6	222 (7.2)	60 (2.0)
7	212 (8.5)	37 (1.5)
8	102 (5.7)	6 (0.3)
9	12 (1.3)	1 (0.1)
10	4 (1.5)	1 (0.4)

*Note:* For % denominator discarded patients where follow-up time did not allow the given number of follow-ups.

**Table 2:** Predictive value of the pre-evaluative “yellow flags” on recovery and satisfaction after the trial period (T1).

Predictors	Recovery			Satisfaction		
	OR	95% CI	Adjusted *p* value°	OR	95% CI	Adjusted *p* value°
PCI Total Active Coping	1.010	[1.001, 1.020]	**0.028**	1.012	[1.002, 1.021]	**0.017**
PCI Pain Transforming	1.004	[0.998, 1.010]	0.404	1.005	[0.999, 1.012]	0.239
PCI Distraction	1.007	[1.000, 1.014]	0.127	1.009	[1.002, 1.017]	**0.043**
PCI Reducing Demands	1.003	[0.998, 1.008]	0.404	1.002	[0.996, 1.007]	0.524
PCI Total Passive Coping	0.996	[0.988, 1.004]	0.372	0.988	[0.980, 0.997]	**0.006**
PCI Retreating	0.998	[0.992, 1.005]	1.000	0.992	[0.985, 0.998]	**0.031**
PCI Worrying	0.996	[0.990, 1.003]	0.871	0.991	[0.984, 0.998]	**0.031**
PCI Resting	0.999	[0.993, 1.006]	1.000	0.997	[0.990, 1.003]	0.303

IAS Illness Anxiety	0.987	[0.971, 1.003]	0.215	0.971	[0.956, 0.987]	**0.001**
IAS Illness Behavior	1.022	[0.984, 1.060]	0.258	0.971	[0.932, 1.010]	0.141

SCL-90-R Psychoneuroticism^∗^	0.998	[0.988, 1.010]	0.782	0.990	[0.979, 1.002]	0.089
SCL-90-R Agoraphobia^∗^	0.998	[0.989, 1.008]	1.000	0.994	[0.985, 1.004]	0.987
SCL-90-R Anxiety^∗^	0.998	[0.989, 1.008]	1.000	0.992	[0.982, 1.001]	0.452
SCL-90-R Depression^∗^	0.995	[0.988, 1.003]	1.000	0.989	[0.981, 0.997]	**0.048**
SCL-90-R Somatic Complaints^∗^	1.010	[1.000, 1.021]	0.300	1.002	[0.992, 1.013]	0.989
SCL-90-R Insufficiency^∗^	0.998	[0.990, 1.006]	1.000	0.996	[0.987, 1.004]	0.989
SCL-90-R Sensitivity^∗^	0.995	[0.985, 1.005]	1.000	0.990	[0.980, 1.000]	0.359
SCL-90-R Hostility^∗^	1.002	[0.991, 1.015]	1.000	0.994	[0.983, 1.006]	0.989
SCL-90-R Sleep Problems^∗^	1.005	[1.000, 1.011]	0.300	1.005	[0.999, 1.011]	0.452

*Note:* OR with 95% CI from the logistic regression model and *p* value (°) corrected for multiple testing using the Bonferroni–Holm correction (3 subscales of PCI Total Active Coping, 3 subscales of PCI Total Passive Coping, 8 subscales of SCL-90-R, and 2 subscales of IAS). Statistically significant *p* values are shown in bold.

^∗^Dutch version and scoring of the SCL-90-R.

**Table 3:** Comparison of psychological pre-evaluative screening measures for the noncomplete group and the complete group (T2).

Variable	*n* T2 noncomplete group	Mean (SD)	*n* T2 complete group	Mean (SD)	Mean difference [95% CI]	Raw *p* value *t*-test°	Adjusted *p* value
SCL-90-R Agoraphobia^∗^	872	10.6 (15.9)	1070	9.5 (14.8)	1.1 [−0.2, 2.5]	0.103	0.694
SCL-90-R Anxiety^∗^	872	18.7 (15.7)	1070	17.8 (15.1)	0.9 [−0.5, 2.3]	0.201	0.805
SCL-90-R Depression^∗^	872	26.1 (19.1)	1070	25.3 (18.4)	0.8 [−0.9, 2.5]	0.361	1
SCL-90-R Somatic Complaints^∗^	872	37.9 (15.5)	1070	36.8 (14.6)	1.1 [−0.2, 2.5]	0.099	0.694
SCL-90-R Insufficiency^∗^	872	35.8 (18.4)	1070	35.2 (18.2)	0.6 [−1.1, 2.2]	0.484	1
SCL-90-R Sensitivity^∗^	872	13.7 (14.7)	1070	12.7 (13.5)	1 [−0.2, 2.3]	0.115	0.694
SCL-90-R Hostility^∗^	872	11.6 (13)	1070	11.4 (12.9)	0.2 [−0.9, 1.4]	0.705	1
SCL-90-R Sleep Problems^∗^	872	59.6 (28.7)	1070	62.2 (27.2)	−2.6 [−5.1, −0.1]	**0.041**	0.327
SCL-90-R Psychoneuroticism^∗^	872	22.9 (13.4)	1070	22.1 (12.9)	0.8 [−0.4, 1.9]	0.206	0.206

PCI Total Active Coping	1265	44.2 (15)	1397	45.2 (14.4)	−1 [−2.1, 0.1]	0.073	0.073
PCI Pain Transforming	1265	37.4 (22.3)	1397	39.7 (21.5)	−2.3 [−4, −0.6]	**0.007**	**0.020**
PCI Distraction	1265	47.8 (19)	1397	49.4 (18.5)	−1.6 [−3, −0.2]	**0.027**	0.054
PCI Reducing Demands	1265	47 (26.8)	1397	45.4 (26)	1.6 [−0.4, 3.7]	0.108	0.108
PCI Total Passive Coping	1265	50 (16.7)	1397	48.1 (16)	1.9 [0.6, 3.1]	**0.004**	**0.004**
PCI Retreating	1265	38.4 (20.7)	1397	36 (19.9)	2.4 [0.9, 4]	**0.002**	**0.006**
PCI Worrying	1265	50.4 (20.3)	1397	49.3 (20.1)	1.1 [−0.5, 2.6]	0.166	0.166
PCI Resting	1265	65.6 (22.1)	1397	63.2 (21.9)	2.5 [0.8, 4.1]	**0.004**	**0.008**

IAS Illness Anxiety	1265	10.2 (8)	1397	9.6 (7.6)	0.6 [0, 1.2]	**0.046**	**0.046**
IAS Illness Behavior	1265	16.9 (3.6)	1397	16.5 (3.3)	0.4 [0.2, 0.7]	**0.002**	**0.004**

*Note:* Statistically significant *p* values are shown in bold.

^∗^Dutch version and scoring of the SCL-90-R.

°For PreIAS Illness Behavior and PreSCL-90-R Sleep Problems, the Welch test is used (unequal variances).

**Table 4:** Comparison of psychological postevaluative measures for the noncomplete group and the complete group (T2).

Variable	*n* T2 noncomplete group	Mean (SD)	*n* T2 complete group	Mean (SD)	Mean difference [95% CI]	Raw *p* value *t*-test°	Adjusted *p* value
SCL-90-R Agoraphobia^∗^	869	6.1 (11.5)	1062	4.3 (8.9)	1.7 [0.8, 2.6]	**< 0.001**	**0.001**
SCL-90-R Anxiety^∗^	869	9.6 (11.3)	1062	7.7 (9.5)	1.9 [1, 2.9]	**< 0.001**	**< 0.001**
SCL-90-R Depression^∗^	869	14.2 (14.5)	1062	11.0 (11.9)	3.2 [2, 4.4]	**< 0.001**	**< 0.001**
SCL-90-R Somatic Complaints^∗^	869	20.2 (14.3)	1062	15.9 (11.9)	4.3 [3.1, 5.5]	**< 0.001**	**< 0.001**
SCL-90-R Insufficiency^∗^	869	20.9 (15.5)	1062	17.5 (14.6)	3.4 [2, 4.7]	**< 0.001**	**< 0.001**
SCL-90-R Sensitivity^∗^	869	7.5 (10.4)	1062	5.9 (8.6)	1.6 [0.7, 2.4]	**< 0.001**	**0.001**
SCL-90-R Hostility^∗^	869	5.9 (8.8)	1062	4.7 (7.2)	1.2 [0.5, 2]	**0.001**	**0.001**
SCL-90-R Sleep Problems^∗^	869	34.5 (28)	1062	29.1 (26.2)	5.4 [2.9, 7.8]	**< 0.001**	**< 0.001**
SCL-90-R Psychoneuroticism^∗^	869	12.5 (10.6)	1062	10.0 (8.9)	2.5 [1.6, 3.4]	**< 0.001**	**< 0.001**

GPE Recovery	1265	5.6 (0.8)	1397	5.8 (0.6)	−0.2 [−0.3, −0.2]	**< 0.001**	**< 0.001**
GPE Satisfaction	1265	5.7 (1.2)	1397	6.1 (0.7)	−0.3 [−0.4, −0.3]	**< 0.001**	**< 0.001**

*Note:* Statistically significant *p* values are shown in bold.

^∗^Dutch version and scoring of the SCL-90-R.

°Welch test with unequal variances.

**Table 5:** Means (SD) of the battery lifetime in days and years across indication type and battery type (rechargeable versus nonrechargeable internal pulse generators), together with median battery survival time and battery survival time to 25% and 75% failures in years.

	No. of records	Mean (SD) (days)	Mean (SD) (years)	Percentiles of survival distribution (years)		
				25% failures	Median	75% failures
Rechargeable FBSS	889	1.922 (1.347)	5.27 (3.69)	1.9	3.6	9.0
Nonrechargeable FBSS	2687	1.753 (1.117)	4.8 (3.06)	2.8	4.1	6.1
Rechargeable FNSS	50	1.555 (1.410)	4.26 (3.86)	1.4	2.4	9.0
Nonrechargeable FNSS	299	1.701 (1.111)	4.66 (3.04)	2.6	4.0	5.9
Rechargeable “Other”	119	2.118 (1.292)	5.80 (3.54)	2.1	5.8	9.1
Nonrechargeable “Other”	354	2.057 (1.069)	5.64 (2.93)	3.5	5.2	7.1

**Table 6:** Difference in battery lifetime in days and years between the three reported indication types (FBSS, FNSS, and “Other”) per battery type and difference between battery type per indication type.

	Difference (in days)	95% CI	Difference (in years)	95% CI	*p* value^∗^
FBSS—FNSS (rechargeable)	371	[−87, 830]	1.02	[−0.24, 2.27]	0.139
FBSS—“Other” (rechargeable)	−192	[−500, 116]	−0.53	[−1.37, 0.32]	0.310
FNSS—“Other” (rechargeable)	−563	[−1095, −32]	−1.54	[−3.00, −0.09]	**0.035**

FBSS—FNSS (nonrechargeable)	56	[−114, 226]	0.15	[−0.31, 0.62]	0.720
FBSS—“Other” (nonrechargeable)	−298	[−453, −143]	−0.82	[−1.24, −0.39]	**< 0.001**
FNSS—“Other” (nonrechargeable)	−354	[−571, −136]	−0.97	[−1.56, −0.37]	**< 0.001**

Rechargeable FBSS vs. nonrechargeable FBSS	77.81	[−7.34, 163.28]	0.21	[−0.02, 0.45]	0.069

Rechargeable FNSS vs. nonrechargeable FNSS	−203.55	[−518.28, 113.44]	−0.56	[−1.42, 0.31]	0.208

Rechargeable “Other” vs. nonrecharageable “Other”	8.11	[−221.95, 239.07]	0.02	[−0.61, 0.65]	0.945

*Note:* Statistically significant *p* values are shown in bold.

^∗^Linear mixed model, *p* value is corrected for multiple testing with Tukey correction for comparing the 3 indication types (FBSS, FNSS, and Other).

## Data Availability

The data that support the findings of this study are available on request from the corresponding author. The data are not publicly available due to privacy or ethical restrictions.

## Supporting Information

The supporting information, Table S1, provides additional information on the results of the various *post hoc* comparisons within the linear mixed model for the outcome measures across all time points (T0–T11). This supporting information is essential not only for supporting the described conclusions but also for presenting more detailed analyses of the linear mixed model.
