# Supplementary material for: Multidisciplinary Approach to Spinal Cord Stimulation for Persistent Spinal Pain Syndromes: A 65-Month Integrated Data Collection From the Belgian Neuro-Pain® Real-World Data Register
Source: Pain Res Manag. 2025 Aug 4;2025:7880611. doi: 10.1155/prm/7880611 (PMC12339138; doi:10.1155/prm/7880611)
Supplement: Supporting Information — Additional supporting information can be found online in the Supporting Information section. [file 7880611.f1.docx]

Supplementary Table S1: Results of the different posthoc comparisons of the linear mixed model of the outcome measures over time (T0 to T11)

| Variable | Comparison | Mean difference | 95% CI | Adjusted P-value^+^ |
| --- | --- | --- | --- | --- |
| SCL90 Agoraphobia* | T1 to T0 | -4.84 | [-5.53,-4.15] | **0.000** |
|  | T2 to T1 | 1.88 | [0.96,2.81] | **0.000** |
|  | T3 to T2 | 0.62 | [-0.82,2.05] | 1.000 |
|  | T4 to T3 | -0.01 | [-1.81,1.78] | 1.000 |
|  | T5 to T4 | 0.70 | [-1.24,2.65] | 1.000 |
|  | T6 to T5 | -0.16 | [-2.31,1.99] | 1.000 |
|  | T7 to T6 | 0.32 | [-2.09,2.74] | 1.000 |
|  | T8 to T7 | -0.22 | [-2.88,2.43] | 1.000 |
|  | T9 to T8 | 1.59 | [-1.37,4.54] | 1.000 |
|  | T10 to T9 | 0.56 | [-3.38,4.49] | 1.000 |
|  | T11 to T10 | -0.90 | [-9.80,8.00] | 1.000 |
| SCL90 Anxiety* | T1 to T0 | -9.39 | [-10.10,-8.68] | **0.000** |
|  | T2 to T1 | 3.40 | [2.45,4.35] | **0.000** |
|  | T3 to T2 | 0.49 | [-0.98,1.97] | 1.000 |
|  | T4 to T3 | 0.75 | [-1.09,2.59] | 1.000 |
|  | T5 to T4 | 0.04 | [-1.96,2.04] | 1.000 |
|  | T6 to T5 | 0.57 | [-1.63,2.78] | 1.000 |
|  | T7 to T6 | -0.06 | [-2.54,2.42] | 1.000 |
|  | T8 to T7 | 0.59 | [-2.14,3.31] | 1.000 |
|  | T9 to T8 | 0.66 | [-2.37,3.70] | 1.000 |
|  | T10 to T9 | 1.54 | [-2.50,5.58] | 1.000 |
|  | T11 to T10 | -5.92 | [-15.06,3.22] | 0.615 |
| SCL90 Depression* | T1 to T0 | -13.07 | [-13.96,-12.17] | **0.000** |
|  | T2 to T1 | 4.32 | [3.12,5.51] | **0.000** |
|  | T3 to T2 | 0.91 | [-0.94,2.77] | 1.000 |
|  | T4 to T3 | 0.37 | [-1.95,2.69] | 1.000 |
|  | T5 to T4 | 0.13 | [-2.40,2.65] | 1.000 |
|  | T6 to T5 | 0.63 | [-2.15,3.41] | 1.000 |
|  | T7 to T6 | 0.84 | [-2.29,3.96] | 1.000 |
|  | T8 to T7 | 0.13 | [-3.31,3.58] | 1.000 |
|  | T9 to T8 | 1.74 | [-2.09,5.57] | 1.000 |
|  | T10 to T9 | 0.41 | [-4.68,5.51] | 1.000 |
|  | T11 to T10 | -2.51 | [-14.04,9.02] | 1.000 |
| SCL90 Somatic complaints* | T1 to T0 | -19.17 | [-20.00,-18.34] | **0.000** |
|  | T2 to T1 | 6.34 | [5.23,7.45] | **0.000** |
|  | T3 to T2 | 1.27 | [-0.46,2.99] | 0.348 |
|  | T4 to T3 | 0.57 | [-1.58,2.72] | 1.000 |
|  | T5 to T4 | 0.47 | [-1.87,2.81] | 1.000 |
|  | T6 to T5 | 0.17 | [-2.41,2.75] | 1.000 |
|  | T7 to T6 | 0.06 | [-2.84,2.96] | 1.000 |
|  | T8 to T7 | 0.60 | [-2.60,3.79] | 1.000 |
|  | T9 to T8 | 0.52 | [-3.04,4.07] | 1.000 |
|  | T10 to T9 | 1.76 | [-2.97,6.49] | 1.000 |
|  | T11 to T10 | -3.27 | [-13.97,7.43] | 1.000 |
| SCL90 Insufficiency* | T1 to T0 | -16.14 | [-17.04,-15.24] | **0.000** |
|  | T2 to T1 | 5.28 | [4.07,6.48] | **0.000** |
|  | T3 to T2 | 1.46 | [-0.41,3.34] | 0.254 |
|  | T4 to T3 | 0.28 | [-2.06,2.62] | 1.000 |
|  | T5 to T4 | 0.58 | [-1.96,3.12] | 1.000 |
|  | T6 to T5 | 0.73 | [-2.07,3.54] | 1.000 |
|  | T7 to T6 | 0.20 | [-2.95,3.35] | 1.000 |
|  | T8 to T7 | -0.92 | [-4.39,2.55] | 1.000 |
|  | T9 to T8 | 2.51 | [-1.35,6.37] | 0.541 |
|  | T10 to T9 | 0.23 | [-4.91,5.37] | 1.000 |
|  | T11 to T10 | -3.10 | [-14.73,8.53] | 1.000 |
| SCL90 Sensitivity* | T1 to T0 | -6.47 | [-7.09,-5.86] | **0.000** |
|  | T2 to T1 | 2.58 | [1.75,3.40] | **0.000** |
|  | T3 to T2 | 0.51 | [-0.78,1.79] | 1.000 |
|  | T4 to T3 | 0.34 | [-1.26,1.94] | 1.000 |
|  | T5 to T4 | 0.04 | [-1.69,1.78] | 1.000 |
|  | T6 to T5 | 0.24 | [-1.68,2.16] | 1.000 |
|  | T7 to T6 | 0.77 | [-1.38,2.93] | 1.000 |
|  | T8 to T7 | -0.55 | [-2.92,1.82] | 1.000 |
|  | T9 to T8 | 2.51 | [-0.13,5.15] | 0.067 |
|  | T10 to T9 | 0.64 | [-2.87,4.15] | 1.000 |
|  | T11 to T10 | -4.08 | [-12.02,3.86] | 1.000 |
| SCL90 Hostility* | T1 to T0 | -5.98 | [-6.58,-5.39] | **0.000** |
|  | T2 to T1 | 1.81 | [1.02,2.60] | **0.000** |
|  | T3 to T2 | 0.19 | [-1.04,1.42] | 1.000 |
|  | T4 to T3 | 0.72 | [-0.82,2.26] | 1.000 |
|  | T5 to T4 | -0.77 | [-2.44,0.90] | 1.000 |
|  | T6 to T5 | 0.45 | [-1.39,2.30] | 1.000 |
|  | T7 to T6 | 0.22 | [-1.85,2.30] | 1.000 |
|  | T8 to T7 | -0.15 | [-2.43,2.13] | 1.000 |
|  | T9 to T8 | 0.77 | [-1.77,3.31] | 1.000 |
|  | T10 to T9 | 0.17 | [-3.21,3.55] | 1.000 |
|  | T11 to T10 | -2.50 | [-10.14,5.15] | 1.000 |
| SCL90 Sleep problems* | T1 to T0 | -29.37 | [-31.07,-27.66] | **0.000** |
|  | T2 to T1 | 8.44 | [6.18,10.71] | **0.000** |
|  | T3 to T2 | 1.69 | [-1.83,5.21] | 1.000 |
|  | T4 to T3 | 0.29 | [-4.12,4.69] | 1.000 |
|  | T5 to T4 | 3.41 | [-1.38,8.19] | 0.367 |
|  | T6 to T5 | -3.85 | [-9.14,1.44] | 0.367 |
|  | T7 to T6 | 1.21 | [-4.74,7.15] | 1.000 |
|  | T8 to T7 | 0.01 | [-6.53,6.55] | 1.000 |
|  | T9 to T8 | 1.30 | [-5.98,8.58] | 1.000 |
|  | T10 to T9 | -2.18 | [-11.86,7.49] | 1.000 |
|  | T11 to T10 | 2.92 | [-19.00,24.83] | 1.000 |
| SCL90 Psychoneuroticism* | T1 to T0 | -11.13 | [-11.75,-10.52] | **0.000** |
|  | T2 to T1 | 3.85 | [3.03,4.68] | **0.000** |
|  | T3 to T2 | 0.76 | [-0.52,2.03] | 0.859 |
|  | T4 to T3 | 0.45 | [-1.14,2.05] | 1.000 |
|  | T5 to T4 | 0.31 | [-1.43,2.04] | 1.000 |
|  | T6 to T5 | 0.22 | [-1.69,2.13] | 1.000 |
|  | T7 to T6 | 0.44 | [-1.71,2.59] | 1.000 |
|  | T8 to T7 | -0.01 | [-2.37,2.35] | 1.000 |
|  | T9 to T8 | 1.51 | [-1.12,4.14] | 0.859 |
|  | T10 to T9 | 0.74 | [-2.76,4.24] | 1.000 |
|  | T11 to T10 | -3.21 | [-11.13,4.71] | 1.000 |
| SCL90 Somatization° | T1 to T0 | -13.92 | [-14.75,-13.09] | **0.000** |
|  | T2 to T1 | 5.60 | [4.43,6.77] | **0.000** |
|  | T3 to T2 | -0.07 | [-3.00,2.86] | 1.000 |
|  | T4 to T3 | 0.73 | [-3.28,4.74] | 1.000 |
|  | T5 to T4 | 0.52 | [-4.17,5.20] | 1.000 |
|  | T6 to T5 | 2.75 | [-2.88,8.37] | 1.000 |
|  | T7 to T6 | -1.72 | [-7.98,4.54] | 1.000 |
|  | T8 to T7 | -1.44 | [-8.65,5.76] | 1.000 |
|  | T9 to T8 | 0.47 | [-7.98,8.92] | 1.000 |
|  | T10 to T9 | 2.98 | [-6.83,12.78] | 1.000 |
|  | T11 to T10 | -2.20 | [-21.42,17.01] | 1.000 |
| SCL90 Obsessive-compulsiveness° | T1 to T0 | -8.97 | [-9.71,-8.23] | **0.000** |
|  | T2 to T1 | 3.27 | [2.22,4.33] | **0.000** |
|  | T3 to T2 | 0.14 | [-2.51,2.79] | 1.000 |
|  | T4 to T3 | 0.53 | [-3.09,4.16] | 1.000 |
|  | T5 to T4 | 0.03 | [-4.21,4.26] | 1.000 |
|  | T6 to T5 | 2.36 | [-2.72,7.45] | 1.000 |
|  | T7 to T6 | -0.97 | [-6.63,4.69] | 1.000 |
|  | T8 to T7 | -2.55 | [-9.06,3.95] | 1.000 |
|  | T9 to T8 | 1.36 | [-6.26,8.97] | 1.000 |
|  | T10 to T9 | -1.33 | [-10.18,7.51] | 1.000 |
|  | T11 to T10 | -0.75 | [-17.98,16.47] | 1.000 |
| SCL90 Sensitivity° | T1 to T0 | -4.94 | [-5.52,-4.35] | **0.000** |
|  | T2 to T1 | 1.86 | [1.03,2.69] | **0.000** |
|  | T3 to T2 | -0.32 | [-2.41,1.78] | 1.000 |
|  | T4 to T3 | 1.42 | [-1.44,4.28] | 1.000 |
|  | T5 to T4 | -1.02 | [-4.36,2.31] | 1.000 |
|  | T6 to T5 | 1.42 | [-2.59,5.43] | 1.000 |
|  | T7 to T6 | -0.58 | [-5.04,3.89] | 1.000 |
|  | T8 to T7 | -1.68 | [-6.81,3.45] | 1.000 |
|  | T9 to T8 | 1.38 | [-4.62,7.37] | 1.000 |
|  | T10 to T9 | 1.25 | [-5.72,8.22] | 1.000 |
|  | T11 to T10 | -0.01 | [-13.58,13.55] | 1.000 |
| SCL90 Depression° | T1 to T0 | -11.97 | [-13.00,-10.94] | **0.000** |
|  | T2 to T1 | 4.60 | [3.14,6.07] | **0.000** |
|  | T3 to T2 | -0.51 | [-4.20,3.18] | 1.000 |
|  | T4 to T3 | 1.28 | [-3.76,6.32] | 1.000 |
|  | T5 to T4 | 0.49 | [-5.39,6.37] | 1.000 |
|  | T6 to T5 | 2.79 | [-4.28,9.85] | 1.000 |
|  | T7 to T6 | -1.78 | [-9.65,6.09] | 1.000 |
|  | T8 to T7 | -2.87 | [-11.92,6.18] | 1.000 |
|  | T9 to T8 | -0.40 | [-10.99,10.18] | 1.000 |
|  | T10 to T9 | 3.47 | [-8.83,15.77] | 1.000 |
|  | T11 to T10 | 0.58 | [-23.39,24.56] | 1.000 |
| SCL90 Anxiety° | T1 to T0 | -6.33 | [-6.96,-5.70] | **0.000** |
|  | T2 to T1 | 2.49 | [1.59,3.39] | **0.000** |
|  | T3 to T2 | -0.10 | [-2.36,2.15] | 1.000 |
|  | T4 to T3 | 0.90 | [-2.18,3.99] | 1.000 |
|  | T5 to T4 | -0.59 | [-4.18,3.01] | 1.000 |
|  | T6 to T5 | 1.76 | [-2.56,6.09] | 1.000 |
|  | T7 to T6 | -0.72 | [-5.53,4.09] | 1.000 |
|  | T8 to T7 | -2.24 | [-7.78,3.29] | 1.000 |
|  | T9 to T8 | -0.44 | [-6.91,6.03] | 1.000 |
|  | T10 to T9 | 3.53 | [-3.99,11.05] | 1.000 |
|  | T11 to T10 | -0.25 | [-14.90,14.40] | 1.000 |
| SCL90 Hostility° | T1 to T0 | -3.27 | [-3.67,-2.87] | **0.000** |
|  | T2 to T1 | 1.09 | [0.52,1.67] | **0.000** |
|  | T3 to T2 | -0.18 | [-1.61,1.26] | 1.000 |
|  | T4 to T3 | 0.62 | [-1.34,2.59] | 1.000 |
|  | T5 to T4 | -0.43 | [-2.72,1.86] | 1.000 |
|  | T6 to T5 | 1.20 | [-1.55,3.95] | 1.000 |
|  | T7 to T6 | -0.50 | [-3.57,2.56] | 1.000 |
|  | T8 to T7 | -0.26 | [-3.78,3.26] | 1.000 |
|  | T9 to T8 | -0.32 | [-4.44,3.81] | 1.000 |
|  | T10 to T9 | 0.35 | [-4.44,5.14] | 1.000 |
|  | T11 to T10 | 2.25 | [-7.10,11.60] | 1.000 |
| SCL90 Phobic Anxiety° | T1 to T0 | -3.07 | [-3.56,-2.59] | **0.000** |
|  | T2 to T1 | 1.54 | [0.85,2.23] | **0.000** |
|  | T3 to T2 | -0.23 | [-1.97,1.50] | 1.000 |
|  | T4 to T3 | 0.94 | [-1.43,3.31] | 1.000 |
|  | T5 to T4 | -0.61 | [-3.37,2.15] | 1.000 |
|  | T6 to T5 | 0.50 | [-2.82,3.83] | 1.000 |
|  | T7 to T6 | -0.64 | [-4.34,3.05] | 1.000 |
|  | T8 to T7 | -1.79 | [-6.04,2.46] | 1.000 |
|  | T9 to T8 | 3.27 | [-1.70,8.23] | 0.577 |
|  | T10 to T9 | 0.03 | [-5.73,5.80] | 1.000 |
|  | T11 to T10 | -0.16 | [-11.36,11.04] | 1.000 |
| SCL90 Paranoid Ideation° | T1 to T0 | -1.89 | [-2.24,-1.54] | **0.000** |
|  | T2 to T1 | 0.62 | [0.12,1.13] | **0.005** |
|  | T3 to T2 | -0.29 | [-1.56,0.98] | 1.000 |
|  | T4 to T3 | 0.87 | [-0.87,2.60] | 1.000 |
|  | T5 to T4 | -0.32 | [-2.34,1.70] | 1.000 |
|  | T6 to T5 | 0.44 | [-1.99,2.87] | 1.000 |
|  | T7 to T6 | -0.26 | [-2.97,2.45] | 1.000 |
|  | T8 to T7 | -1.06 | [-4.17,2.05] | 1.000 |
|  | T9 to T8 | -0.06 | [-3.70,3.57] | 1.000 |
|  | T10 to T9 | 0.44 | [-3.78,4.67] | 1.000 |
|  | T11 to T10 | -0.38 | [-8.60,7.84] | 1.000 |
| SCL90 Psychoticism° | T1 to T0 | -2.98 | [-3.41,-2.55] | **0.000** |
|  | T2 to T1 | 1.00 | [0.39,1.61] | **0.000** |
|  | T3 to T2 | -0.22 | [-1.76,1.32] | 1.000 |
|  | T4 to T3 | 1.21 | [-0.89,3.32] | 0.947 |
|  | T5 to T4 | -0.70 | [-3.15,1.76] | 1.000 |
|  | T6 to T5 | 1.12 | [-1.83,4.07] | 1.000 |
|  | T7 to T6 | -0.78 | [-4.06,2.50] | 1.000 |
|  | T8 to T7 | -1.22 | [-5.00,2.55] | 1.000 |
|  | T9 to T8 | 0.37 | [-4.04,4.78] | 1.000 |
|  | T10 to T9 | 0.37 | [-4.76,5.49] | 1.000 |
|  | T11 to T10 | 0.41 | [-9.55,10.37] | 1.000 |
| SCL90 Global Severity Index° | T1 to T0 | -0.71 | [-0.76,-0.65] | **0.000** |
|  | T2 to T1 | 0.27 | [0.20,0.35] | **0.000** |
|  | T3 to T2 | -0.03 | [-0.23,0.17] | 1.000 |
|  | T4 to T3 | 0.11 | [-0.17,0.38] | 1.000 |
|  | T5 to T4 | -0.03 | [-0.34,0.29] | 1.000 |
|  | T6 to T5 | 0.16 | [-0.22,0.54] | 1.000 |
|  | T7 to T6 | -0.08 | [-0.51,0.34] | 1.000 |
|  | T8 to T7 | -0.18 | [-0.67,0.30] | 1.000 |
|  | T9 to T8 | 0.06 | [-0.51,0.63] | 1.000 |
|  | T10 to T9 | 0.13 | [-0.53,0.79] | 1.000 |
|  | T11 to T10 | -0.01 | [-1.30,1.28] | 1.000 |
| PCI Distraction | T2 to T0 | 4.02 | [2.70,5.34] | **0.000** |
|  | T3 to T2 | -0.72 | [-2.92,1.48] | 1.000 |
|  | T4 to T3 | -1.86 | [-4.66,0.93] | 0.575 |
|  | T5 to T4 | -0.87 | [-3.92,2.18] | 1.000 |
|  | T6 to T5 | 0.69 | [-2.69,4.07] | 1.000 |
|  | T7 to T6 | 0.98 | [-2.80,4.76] | 1.000 |
|  | T8 to T7 | -1.46 | [-5.64,2.72] | 1.000 |
|  | T9 to T8 | -0.28 | [-4.95,4.39] | 1.000 |
|  | T10 to T9 | -1.01 | [-7.14,5.12] | 1.000 |
|  | T11 to T10 | 5.78 | [-7.91,19.46] | 1.000 |
| PCI Reducing demands | T2 tot T0 | -5.63 | [-7.45,-3.81] | **0.000** |
|  | T3 to T2 | -0.13 | [-3.17,2.90] | 1.000 |
|  | T4 to T3 | 0.24 | [-3.64,4.11] | 1.000 |
|  | T5 to T4 | 0.21 | [-4.03,4.44] | 1.000 |
|  | T6 to T5 | -0.22 | [-4.92,4.48] | 1.000 |
|  | T7 to T6 | 0.26 | [-5.00,5.52] | 1.000 |
|  | T8 to T7 | -2.86 | [-8.68,2.95] | 1.000 |
|  | T9 to T8 | 2.25 | [-4.25,8.74] | 1.000 |
|  | T10 to T9 | -1.24 | [-9.76,7.28] | 1.000 |
|  | T11 to T10 | 2.15 | [-16.93,21.23] | 1.000 |
| PCI Total Passive Coping | T2 tot T0 | -10.73 | [-11.74,-9.72] | **0.000** |
|  | T3 to T2 | 0.55 | [-1.12,2.22] | 1.000 |
|  | T4 to T3 | 0.41 | [-1.71,2.53] | 1.000 |
|  | T5 to T4 | 0.55 | [-1.76,2.85] | 1.000 |
|  | T6 to T5 | 0.29 | [-2.26,2.84] | 1.000 |
|  | T7 to T6 | -0.12 | [-2.97,2.74] | 1.000 |
|  | T8 to T7 | 0.36 | [-2.79,3.52] | 1.000 |
|  | T9 to T8 | -0.48 | [-4.00,3.05] | 1.000 |
|  | T10 to T9 | -2.22 | [-6.85,2.41] | 1.000 |
|  | T11 to T10 | 0.54 | [-9.77,10.86] | 1.000 |
| PCI Retreating | T2 tot T0 | -6.89 | [-8.08,-5.70] | **0.000** |
|  | T3 to T2 | -0.18 | [-2.15,1.79] | 1.000 |
|  | T4 to T3 | 1.28 | [-1.22,3.77] | 1.000 |
|  | T5 to T4 | 0.50 | [-2.22,3.22] | 1.000 |
|  | T6 to T5 | -0.03 | [-3.04,2.98] | 1.000 |
|  | T7 to T6 | 0.79 | [-2.58,4.16] | 1.000 |
|  | T8 to T7 | 0.28 | [-3.44,4.00] | 1.000 |
|  | T9 to T8 | 0.15 | [-4.01,4.30] | 1.000 |
|  | T10 to T9 | -3.40 | [-8.86,2.06] | 0.749 |
|  | T11 to T10 | 0.39 | [-11.78,12.55] | 1.000 |
| PCI Worrying | T2 tot T0 | -13.38 | [-14.67,-12.10] | **0.000** |
|  | T3 to T2 | 1.18 | [-0.95,3.30] | 1.000 |
|  | T4 to T3 | -0.83 | [-3.53,1.86] | 1.000 |
|  | T5 to T4 | 1.61 | [-1.33,4.54] | 1.000 |
|  | T6 to T5 | 0.03 | [-3.22,3.28] | 1.000 |
|  | T7 to T6 | 0.29 | [-3.35,3.92] | 1.000 |
|  | T8 to T7 | 0.25 | [-3.77,4.26] | 1.000 |
|  | T9 to T8 | -0.76 | [-5.25,3.72] | 1.000 |
|  | T10 to T9 | -2.41 | [-8.30,3.49] | 1.000 |
|  | T11 to T10 | 2.37 | [-10.77,15.51] | 1.000 |
| PCI Resting | T2 tot T0 | -11.34 | [-12.76,-9.91] | **0.000** |
|  | T3 to T2 | 0.27 | [-2.10,2.63] | 1.000 |
|  | T4 to T3 | 1.34 | [-1.66,4.34] | 1.000 |
|  | T5 to T4 | -1.19 | [-4.45,2.08] | 1.000 |
|  | T6 to T5 | 1.16 | [-2.46,4.78] | 1.000 |
|  | T7 to T6 | -2.15 | [-6.20,1.90] | 1.000 |
|  | T8 to T7 | 0.87 | [-3.61,5.35] | 1.000 |
|  | T9 to T8 | -1.18 | [-6.18,3.82] | 1.000 |
|  | T10 to T9 | -0.02 | [-6.58,6.55] | 1.000 |
|  | T11 to T10 | -1.52 | [-16.16,13.13] | 1.000 |
| PDI | T3 to T2 | 2.57 | [0.63,4.51] | **0.003** |
|  | T4 to T3 | 0.07 | [-2.35,2.48] | 1.000 |
|  | T5 to T4 | 0.28 | [-2.32,2.88] | 1.000 |
|  | T6 to T5 | -0.41 | [-3.26,2.45] | 1.000 |
|  | T7 to T6 | -0.36 | [-3.56,2.84] | 1.000 |
|  | T8 to T7 | 1.08 | [-2.45,4.62] | 1.000 |
|  | T9 to T8 | 0.68 | [-3.27,4.64] | 1.000 |
|  | T10 to T9 | 1.50 | [-3.71,6.72] | 1.000 |
|  | T11 to T10 | -1.47 | [-12.87,9.92] | 1.000 |
| GPE Recovery | T2 to T1 | -0.43 | [-0.50,-0.36] | **0.000** |
|  | T3 to T2 | -0.10 | [-0.21,0.02] | 0.165 |
|  | T4 to T3 | -0.05 | [-0.20,0.09] | 1.000 |
|  | T5 to T4 | 0.01 | [-0.15,0.17] | 1.000 |
|  | T6 to T5 | -0.07 | [-0.24,0.11] | 1.000 |
|  | T7 to T6 | -0.03 | [-0.23,0.17] | 1.000 |
|  | T8 to T7 | -0.09 | [-0.31,0.13] | 1.000 |
|  | T9 to T8 | -0.15 | [-0.39,0.10] | 0.793 |
|  | T10 to T9 | 0.04 | [-0.28,0.37] | 1.000 |
|  | T11 to T10 | 0.16 | [-0.57,0.89] | 1.000 |
| GPE Satisfaction | T2 tot T1 | -0.64 | [-0.72,-0.57] | **0.000** |
|  | T3 to T2 | 0.00 | [-0.13,0.12] | 1.000 |
|  | T4 to T3 | 0.00 | [-0.16,0.16] | 1.000 |
|  | T5 to T4 | 0.03 | [-0.14,0.21] | 1.000 |
|  | T6 to T5 | -0.06 | [-0.26,0.13] | 1.000 |
|  | T7 to T6 | 0.02 | [-0.19,0.24] | 1.000 |
|  | T8 to T7 | -0.09 | [-0.33,0.15] | 1.000 |
|  | T9 to T8 | 0.00 | [-0.27,0.26] | 1.000 |
|  | T10 to T9 | 0.06 | [-0.29,0.41] | 1.000 |
|  | T11 to T10 | -0.28 | [-1.06,0.51] | 1.000 |

Mean difference with 95% CI and p-value (+) corrected for multiple testing using the Bonferroni Holm correction

(*) Dutch version and scoring of the SCL-90

(°) French and German version and scoring of the SCL-90
